# Gain-of-function variants in GSDME cause pyroptosis and apoptosis associated with post-lingual hearing loss

**DOI:** 10.1007/s00439-024-02694-x

**Published:** 2024-07-27

**Authors:** Yun Xiao, Lei Chen, Kaifan Xu, Meijuan Zhou, Yuechen Han, Jianfen Luo, Yu Ai, Mingming Wang, Yu Jin, Ruifeng Qiao, Shuhui Kong, Zhaomin Fan, Lei Xu, Haibo Wang

**Affiliations:** 1 Department of Otolaryngology-Head and Neck Surgery, Shandong Provincial ENT Hospital, Shandong University, Jinan, 250022 Shandong China; 2 Auditory Implant Center, Shandong Provincial ENT Hospital, Jinan, Shandong China; 3 Clinical Audiology Center, Shandong Provincial ENT Hospital, Jinan, Shandong China; 4 Hearing and Balance Biomedical Engineering Laboratory, Shandong Provincial ENT Hospital, Jinan, Shandong China; 5 Shandong Institute of Otorhinolaryngology, Jinan, Shandong China

## Abstract

Gasdermin E (GSDME), a member of the gasdermin protein family, is associated with post-lingual hearing loss. All GSDME pathogenic mutations lead to skipping exon 8; however, the molecular mechanisms underlying hearing loss caused by GSDME mutants remain unclear. GSDME was recently identified as one of the mediators of programmed cell death, including apoptosis and pyroptosis. Therefore, in this study, we injected mice with GSDME mutant (MT) and examined the expression levels to assess its effect on hearing impairment. We observed loss of hair cells in the organ of Corti and spiral ganglion neurons. Further, the N-terminal release from the GSDME mutant in HEI-OC1 cells caused pyroptosis, characterized by cell swelling and rupture of the plasma membrane, releasing lactate dehydrogenase and cytokines such as interleukin-1β. We also observed that the N-terminal release from GSDME mutants could permeabilize the mitochondrial membrane, releasing cytochromes and activating the mitochondrial apoptotic pathway, thereby generating possible positive feedback on the cleavage of GSDME. Furthermore, we found that treatment with disulfiram or dimethyl fumarate might inhibit pyroptosis and apoptosis by inhibiting the release of GSDME-N from GSDME mutants. In conclusion, this study elucidated the molecular mechanism associated with hearing loss caused by GSDME gene mutations, offering novel insights for potential treatment strategies.

**Supplementary Information:**

The online version contains supplementary material available at 10.1007/s00439-024-02694-x.

## Introduction

Hearing loss (HL) is one of the most prevalent sensory deficits, and thus an important public health problem (Chadha et al. 2021). HL affects the ability to communicate and alters the social lives of patients. Moreover, it is associated with dementia and depression in adults (Li et al. 2014; Lin et al. 2013). Damage to any part of the central or peripheral auditory system can cause HL, which is typically categorized as conductive (damage to middle or outer ear), sensorineural (cochlear or auditory nerve disorder), or mixed (has both conductive and sensorineural components) (Cunningham andTucci. 2017).

Sensorineural HL, which can be classified as prelingual or post-lingual based on the onset before or after language development (average age, 6 years)(Ahmadmehrabi et al. 2021), results from a dysfunction of the organ of Corti (Wagner andShin. 2019), stria vascularis (Wangemann. 2006), or spiral ganglion neurons (Nayagam et al. 2011). The development of sensorineural HL is mainly associated with aging, ototoxic drug exposure, noise exposure, chronic diseases, and genetic mutations (Cunningham andTucci. 2017). Adults are rarely clinically tested for genetic etiology of adult-onset HL because the literature and clinical practice typically emphasize environmental risk factors (Ahmadmehrabi et al. 2021). In recent years, the rapid development of sequencing technologies has led to the identification of genes associated with post-lingual HL. Over 50 post-lingual HL genes have been identified, most of which are inherited in an autosomal dominant pattern (Ahmadmehrabi et al. 2021).

Gasdermin E (*GSDME*), also known as *DFNA5*, was first described as a non-syndromic post-lingual HL gene in 1995 (van Camp et al. 1995). The deafness caused by this gene mutation (OMIM #600,994), which is inherited in an autosomal dominant pattern, is characterized as late-onset and progressive. The *GSDME* gene encodes the GDMSE protein located on human chromosome 7015, which contains 496 amino acids. *GSDME* was classified as a member of the gasdermin family in 2007 (Tamura et al. 2007), which includes *GSDMA*, *GSDMB*, *GSDMC*, *GSDMD*, *GSDME*, and *PJVK* (also known as DFNB59) (Ding et al. 2016; Kayagaki et al. 2015; Rogers et al. 2017). Gasdermins, except for PJVK, possess the pore-forming gasdermin-N domain, which is normally masked by their gasdermin-C domains, conferring them the ability to cause pyroptosis—a necrotic form of programmed cell death (Kovacs andMiao. 2017). When the connection between these two domains is cleaved, plasma membrane oligomers are formed by gasdermin-N domains, ultimately triggering pyroptosis (Ding et al. 2016; Kayagaki et al. 2015; Rogers et al. 2017; Shi et al. 2017). Unlike apoptosis, pyroptosis is an inflammatory form of cell death characterized by cell swelling, pore formation, and rupture of the plasma membrane, resulting in the release of intracellular contents, such as lactate dehydrogenase (LDH) and cytokines including interleukin (IL)-1β and IL-18, and ultimately leading to inflammation (Shi et al. 2017). Additionally, GSDME can also stimulate caspase-3/7 activation and apoptosis by targeting the mitochondria and releasing Cytochrome C (Cyt C)(Rogers et al. 2019).

To date, more than ten mutations of GSDME have been identified and associated with post-lingual HL (Bischoff et al. 2004; Cheng et al. 2007; Li-Yang et al. 2015; Nishio et al. 2014; Park et al. 2010; Van Laer et al. 1998; Yu et al. 2003), many of which are located in exon 8 or flanking sequences of exon 8. Splice-site variations result in the skipping of exon 8 at the mRNA level, resulting in a frameshift and prematurely truncated proteins (Croes et al. 2015; Wang et al. 2018). A previous study reported that cell mortality increases after transfecting the mutant GSDME into yeast and mammalian cells, suggesting that the toxic effect results from gain-of-function mutations (Van Laer et al. 2004). Although GSDME mutations may trigger pyroptosis, their effects on hearing are unclear.

Therefore, the aim of this study was to elucidate the mechanisms of HL caused by *GSDME* mutations at the organism and cellular levels and provide insight into new therapeutic strategies to mitigate the cytotoxicity of GSDME mutation-driven hearing impairment.

## Methods

### Participant recruitment and clinical examinations

Four families with post-lingual HL from the Shandong Provincial ENT Hospital were enrolled in this study. All participants received audiometric evaluations and auditory tests, including pure tone audiometry, tinnitus examination, auditory brainstem response (ABR), and distortion product otoacoustic emission (DPOAE), which were carried out according to standards protocols outlined in our previous study (Zhang et al. 2016).

### Identification of the GSDME variations

The pathogenic variations in *GSDME* were identified using whole-exome sequencing (Yinfeng Gene Technology Co. Ltd., Jinan, China) of samples from families with post-lingual HL. Polymerase chain reaction (PCR) and Sanger sequencing were performed on all samples to determine whether the potential variations in *GSDME* were segregated with the disease phenotype in these families. PCR was performed with the forward primer 5′-GGCCAGGTTCAGCTTACTGT-3′ and reverse primer 5′-TGCCTCCCAGCCTAGTACAT-3′. Direct PCR products were sequenced by Sangon Biotech Co. Ltd. (Qingdao, China).

### Reverse transcription PCR

The total RNA from patient SD4193 and one healthy control from Family 707 were extracted from whole blood samples using the QIAamp RNA Blood Mini Kit (Qiagen, Hilden, Germany) according to manufacturer instructions. Reverse transcription reaction was performed using the Revert Aid First Strand cDNA Synthesis Kit (Thermo Fisher Scientific, Waltham, MA, USA) according to the manufacturer’s instructions. PCR was then carried out using the GSDME-specific primers (forward: 5′-TCTTTCGAGAGTTTGCATTC-3′, reverse: 5′-TTCAGGGGAGTCAAGGTTGG-3′), and the PCR products were sequenced by Sangon Biotech Co., Ltd. (Qingdao, China).

### Plasmid preparation

The cDNAs encoding human *GSDME-WT* or *GSDME-MT* were cloned into the pcDNA3.1-3×Flag plasmid (Shandong Gene&Bio Co. Ltd., Jinan, China). The information regarding the vector is shown in Fig. S1. The lengths of GSDME-WT and GSDME-MT were 1491 bp (NM_004403) and 1298 bp, respectively, without exon 8.

### Mice and plasmid vectors delivered using round window injection

C57BL/6 mice were obtained from Jackson Laboratories (Bar Harbor, ME, USA). Animal experiments adhered to ethical standards, and every attempt was taken to reduce the number of mice utilized and their suffering. Postnatal day 0 (P0) was used to count the days since birth.

The plasmids (*GSDME-WT* or *GSDME-MT)* were mixed with in vivo-jetPEI (PolyPlus, Illkirch-Graffenstaden, France) and 10% glucose solution according to the manufacturer’s protocols. The mixture was incubated for 15 min at room temperature (20–26℃) before injection. The plasmid vectors were delivered into the cochlea through round window injection following a previously published surgical procedure (Gao et al. 2018). Briefly, P7 mice were anesthetized at a low temperature in an ice bath for 5–7 min until loss of consciousness. The mice were then placed on an ice pad for subsequent surgical procedures. Surgery was performed only on the left ear of each animal. Cochleostomy was performed through a post-auricular incision to expose the otic bulla. The round window was identified before injection according to the anatomic landmarks, including the stapedial artery and tympanic ring. Injections were performed through the round window with glass micropipettes (∼ 10 μm) pulled with a micropipette puller (PC-10; Narishige, Japan) and controlled using a micromanipulator (WPI, USA). The volume of the injected materials was controlled at approximately 1.5 µL (including 300 ng of plasmids and 0.05 µL of the in vivo-jetPEI reagent) per cochlea within 1 min. The injected materials in the blank group were replaced with an equal volume of sterile water instead of plasmids. After the injection, the skin incision was conglutinated using a 3 M Vetbond tissue adhesive (3 M, Saint Paul, MN, USA). The mice were subsequently returned to the incubator at 37 °C for 10 min and then returned to their mothers for continued nursing.

### Auditory functional tests

ABR and DPOAE recordings were measured in a soundproof chamber, as described previously (Gao et al. 2018; Liu et al. 2021). The mice were anesthetized with xylazine (10 mg/kg) and ketamine (50 mg/kg), and their body temperatures were maintained at 37 °C using a heating pad during the measurement. The closed-field ABR and DPOAE were recorded based on the TDT system III (Tucker Davis Technologies, Alachua, FL, USA), and changes in the electrical activity of the brain in response to sound were recorded via electrodes. Tone bursts at various stimulus frequencies (4, 8, 12, 16, 24, and 32 kHz) were used to elicit the ABRs. The sound level was decreased from 90 to 20 dB sound pressure levels in 5 dB steps (decibels SPL). The acquired response signal was amplified (10,000×), filtered (0.1–3 kHz), averaged (1024 responses), and run through the BioSigRZ system (Tucker Davis Technologies). The lowest SPL level at which any wave could be distinguished was referred to as the threshold. The hearing threshold was assessed only in the left ear, with the right ear masked by earplugs. For DPOAE measurements, the cubic distortion product was recorded at 2f1-f2 in response to two primary tones, with f2 being equal to the frequencies used in ABR testing, f2/f1 = 1.2, and the f2 level being 10 dB below the f1 level. For each f2/f1 primary pair, the primaries were varied in 5 dB increments from 20 dB SPL to 80 dB SPL (for f2). The threshold of DPOAE was defined as a peak at 2f2-f1, 5 dB above the noise floor.

### Cell cultures and treatments

HEI-OC1 cell lines were cultured in Dulbecco’s modified Eagle’s medium (Gibco, Thermo Fisher Scientific) supplemented with 10% fetal bovine serum at 33 °C in a 10% CO_2_ incubator (Kalinec et al. 2003). Next, 3 × 10^5^ HEI-OC1 cells per well were seeded overnight in 6-well plates for the transfection experiments. Following the manufacturer’s instructions, 2.5 g of plasmid DNA was transfected into the cells the following day using PEIpro Transfection Reagent (PolyPlus). For drug protection experiments, HEI-OC1 cells were treated with disulfiram (1 µM or 5 µM) or dimethyl fumarate (DMF; 20 µM or 50 µM) for 2 h, which was then removed, before transfection. Cells treated with 0.1% dimethyl sulfoxide served as the control group. To examine the morphology of pyroptotic cells, the bright-field cell images were captured using a Leica microscope (Leica, Wetzlar, Germany). The assays of apoptosis and LDH release were performed 24 h after transfection. Finally, culture supernatants and cells were collected 48 h after transfection for western blot analysis.

### Immunofluorescence staining

Four weeks after round window injection, cochleae were harvested and fixed by immersion in 4% paraformaldehyde for 2 h at room temperature (20–26℃) and then treated with 0.5 M ethylenediamine tetra acetic acid solution for 2 h. The basal, middle, and apical turns of the organ of Corti were dissected under a microscope, permeabilized with 1% Triton X-100 in phosphate-buffered saline (PBS) for 30 min, and then washed thrice with 3% bovine serum albumin (BSA) in PBS for 5 min each, followed by blocking with 5% inactivated Donkey Serum, 1% BSA, 0.1% Triton X-100 and 0.02% sodium azide in PBS for 1 h. Samples were then incubated with primary antibodies against MYO7A (1:1000 dilution, 25-6790; Proteus Technologies, Austin, TX, USA) at 4 ℃ overnight. The next day, the primary antibody was removed, and the cells were washed three times with 3% BSA in PBS for 5 min each time. The secondary antibodies against DAPI (1:1000 dilution, D9542; Sigma-Aldrich, St. Louis, MO, USA) and donkey anti-rabbit Alex488 (1:1000 dilution, A21206; Thermo Fisher Scientific) were then added, and the mixture was incubated for 1 h at room temperature in the dark. Sections were blocked and observed under a laser scanning confocal microscope (Leica) after removing the secondary antibody and washing three times with 3% BSA in PBS for 5 min each.

### Hematoxylin and eosin staining

Dissected cochleae were fixed for 2 h in 4% PFA and then decalcified in 0.5 M ethylenediaminetetraacetic acid for 2 h at room temperature. Decalcified cochleae were immersed in 20% sucrose solution for 2 h, then 30% sucrose solution overnight, then embedded in an OCT compound. The embedded cochleae were sectioned on 10 μm slides using a cryostat microtome. The slices were placed in distilled water for 3 min, then stained with hematoxylin for 3 min, washed with distilled water for 3 min, incubated with 1% hydrochloric acid alcohol for 30 s, and washed with distilled water for 10 min. Subsequently, the slices were stained with eosin for 4 min, washed with distilled water for 10 min, dehydrated in graded ethanol (75%, 80%, 90%, and 100%), immersed in xylene solution, and cover-slipped with neutral gum. Images were obtained using a microscope (Olympus BX53; Olympus Co., Tokyo, Japan).

### LDH release assay

Pyroptosis was assessed by detecting the activity of LDH released into cell culture supernatants after various treatments and transfections, using an LDH assay kit (C0017; Beyotime, Shanghai, China) according to the manufacturer’s protocol. An absorbance measurement at 490 nm was performed using a microplate reader (ELx800; Bio Tek, Winooski, VT, USA).

### Flow cytometry

Apoptosis and pyroptosis of treated HEI-OC1 cells were detected through flow cytometry. Annexin V can recognize phosphatidylserine exposed on the external leaflet of the plasma membrane in apoptotic cells as well as stain pyroptotic cells owing to the rupture of the plasma membrane during pyroptosis, which enables it to recognize phosphatidylserine on the inner leaflet. Cells were collected, washed twice with PBS, and stained with the Annexin V-FITC/PI Apoptosis Detection Kit (C1062M; Beyotime). Stained cells were analyzed using a BD Accuri C6 Plus Flow Cytometer (BD Biosciences, Franklin Lakes, NJ, USA) and data were processed using FlowJo software.

### Terminal deoxynucleotidyl transferase dUTP nick end labeling (TUNEL) staining

TUNEL staining was performed to observe apoptosis. HEI-OC1 cells (≈ 6 × 10^4^) were plated on glass bottom cell culture dishes (*Φ* = 15 mm) the day prior to the transfection of *GSDME-MT* plasmids with the PEIpro Transfection Reagent (PolyPlus). TUNEL assay was performed using the Click-iT™ Plus TUNEL assay kit according to the manufacturer’s protocols.

### Western blot analysis

Treated and untreated HEI-OC1 cells were lysed with RIPA lysis buffer (P0013B, Beyotime, China) supplemented with protease inhibitor cocktail (P8340; Sigma-Aldrich) for 30 min on ice, then centrifuged at 12,000 x g for 20 min at 4 °C. The collected supernatants were fractionated via SDS–PAGE and transferred to PVDF membranes (IPVH00010l MilliporeSigma, Burlington, MA, USA). Membranes were blocked with 5% skim milk for 2 h at room temperature and then incubated at 4 °C overnight with primary antibodies diluted in Immunol Staining Primary Antibody Dilution Buffer (P0023A; Beyotime). Subsequently, the membranes were incubated with secondary antibodies for 1 h at room temperature. The signals were detected using Immobilon^®^ Western Chemiluminescent HRP Substrate (P90720; MilliporeSigma). To examine IL-1β release, the collected cell culture supernatants were filtered using 0.22 μm micron filters, then centrifuged at 3,000 x g for 10 min to remove pellet cells and cell debris. Supernatants were transferred to centrifugal filter tubes (UFC800324; MilliporeSigma) and centrifuged at 5,000 × *g* until they reached a concentration of approximately 100 µL. Subsequently, western blot analysis was conducted for the prepared proteins. The primary antibodies used were as follows: GSDME (ab215191; Abcam, Cambridge, UK), Cyt C (11,940 S; Cell Signaling Technology [CST], Danvers, MA, USA), IL-1β (12,242 S; CST), Cleaved Caspase 3 (9661 S; CST), Monoclonal ANTI-FLAG (F3165; Sigma-Aldrich), and β-actin (20536-1-AP; Proteintech, Rosmont, IL, USA).

### Statistical analysis

GraphPad Prism 8.0 software (GraphPad, La Jolla, CA, USA) was used for statistical analysis, and measurement data are presented as mean or mean ± SEM. Student’s *t*-test was used to compare differences between two groups, and one-way analysis of variance (ANOVA) was used to compare differences between two or more groups. A value of *p* < 0.05 was considered statistically significant.

## Results

### Clinical features and identification of the GSDME mutations

Four families with post-lingual HL with an autosomal dominant inheritance pattern were enrolled in this study (Fig. S2a). The participants exhibited symmetrical and bilateral HL with no related syndromic symptoms, as identified through the examination of medical histories or physical examination. The pure tone audiometry results of the affected probands are shown in Fig. S2b, which shows the different degrees of HL. Although there was no continuous tracking of hearing tests, all affected patients had late-onset progressive bilateral HL, and the onset age varied from 9 to 30 years, according to the participants’ questionnaires. The known pathogenic mutations c.991 − 15_991-13delTTC and c.1183 + 4 A > G of *GSDME* were identified in the probands through whole-exome sequencing. Sanger sequencing confirmed that the c.991 − 15_991-13delTTC mutation was segregated with the disease phenotype in families F439, F683, and F578. The c.1183 + 4 A > G mutation was segregated with the phenotype in family F707 (Fig. S2c).

**Fig. 1:**
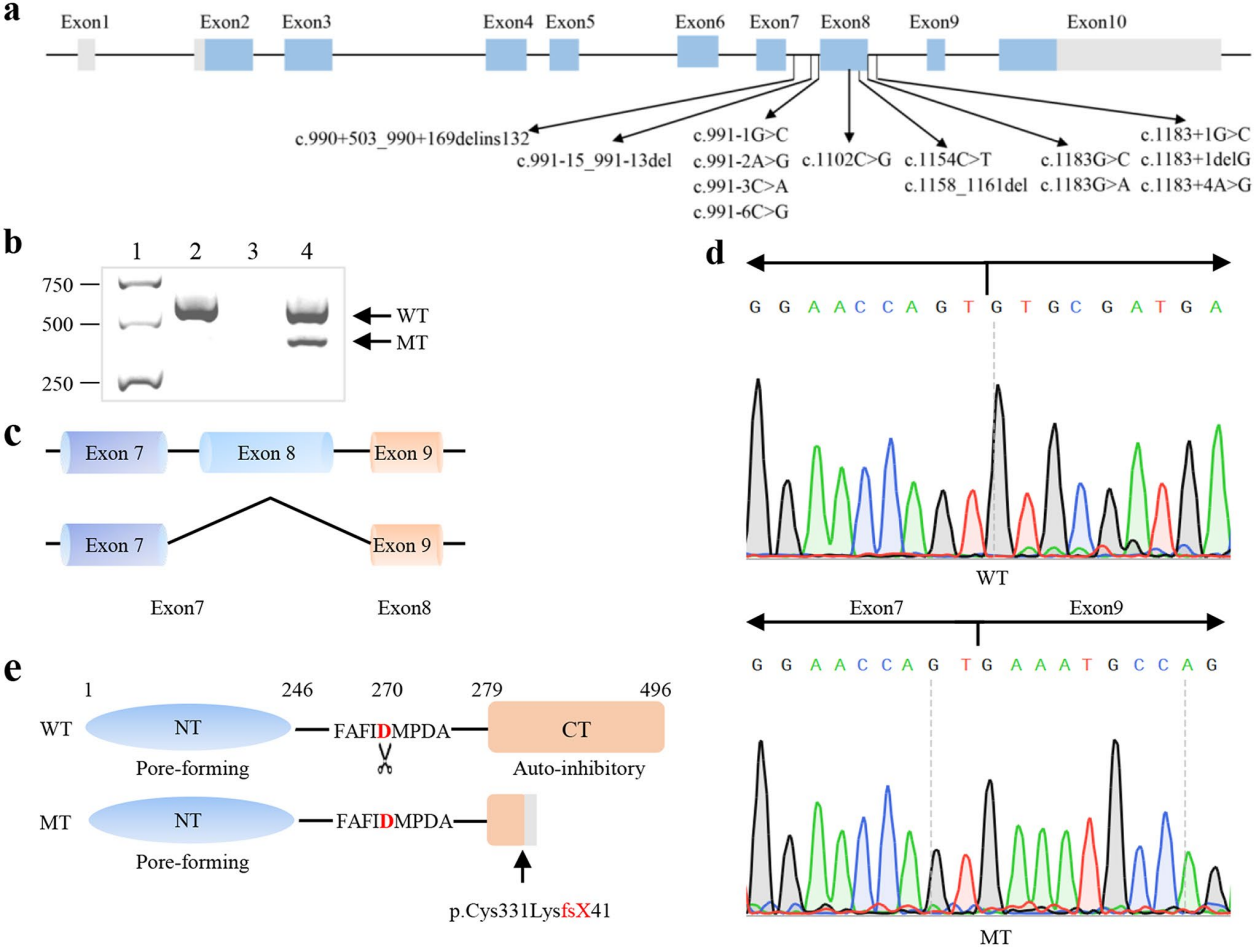
Reported *GSDME* gene variations associated with hearing loss and reverse transcriptase PCR analysis of c.1183 + 4 A > G variation. **(a)** Schematic illustration of the *GSDME* gene showing the positions of all reported mutations associated with post-lingual HL. (**b**) Gel electrophoresis of the cDNA products from the proband SD4193 (lane 4) and a control individual from family F702 (lane 2). Lane 1: molecular weight markers, lane 3: control PCR without a template. The top band (WT) indicates the presence of the wild-type products, including exon8 (543 bp), and the bottom band (MT) represents the mutant products with no exon8 (350 bp). (**c**) Schematic illustration of splicing affected by *GSDME* mutation. (**d**) Sanger sequencing electropherograms of the cSDNA products showing the skipping of exon 8 in the control (WT) and the patient SD4193 (MT). (**e**) Schematic illustration showing the truncated protein resulted from the skipping of exon 8 by *GSDME* mutant

The mutations c.991 − 15_991-13delTTC and c.1183 + 4 A > G of GSDME were located in the flanking sequences of exon 8 (Fig. 1a). A previous study demonstrated that the c.991 − 15_991-13delTTC mutation could result in the skipping of exon 8 at the mRNA level (Wang et al. 2018). Accordingly, the amplification of the cDNA from patient SD4193 identified an additional 350 bp (bp) fragment, supporting the fact that mutation c.1183 + 4 resulted in the splicing defect (Fig. 1b). Sanger sequencing of the 350 bp fragments showed the skipping of exon 8 (Fig. 1c, d), which resulted in the truncated protein with a shorter autoinhibitory domain (Fig. 1e).

### *GSDME-MT* injection in mice results in hearing impairment

**Fig. 2:**
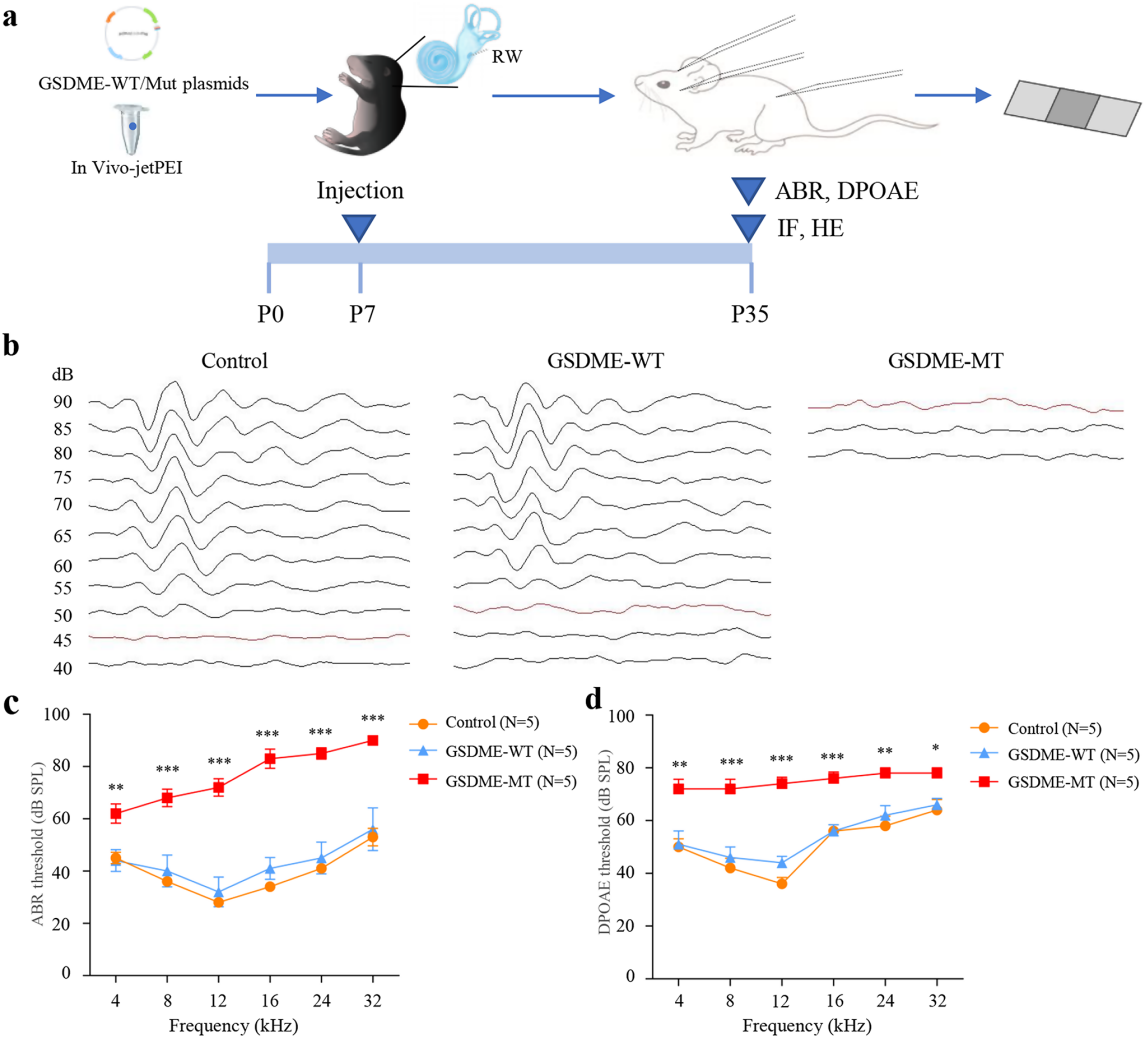
*GSDME-MT* plasmids injection caused hearing impairment in mice. (**a**) Schematic of the experimental setup of the plasmid injection to the cochlea of the mice to investigate the pathogenicity of GSDME gene mutation. (**b**-**d**) Auditory functional tests were performed four weeks after the injection. Representative click ABR waveforms, including (**b**), ABR (**c**), and DPOAE (**d**) thresholds, are plotted. Mice injected with no plasmids were used as the control group. Mice injected with *GSDME-MT* plasmids had significantly higher thresholds of ABR and DPOAE in all frequencies than those of the control group. MT, mutant ABR, auditory brainstem response; DPOAE, distortion product otoacoustic emission DPOAE. Data are shown as mean ± SEM; (*p < 0.05, **p < 0.01, ***p < 0.001)

To investigate the effect of *GSDME* mutation on hearing, the expression vector plasmids of human wild-type *GSDME* (*GSDME-WT*) and *GSDME-MT* were cloned and delivered to the cochleae of P7 wild-type C57BL/6 mice through round window injection (Fig. 2a). To the best of our knowledge, this study is the first time to apply the in vivo-jetPEI to the cochlea. We further investigated the safety of this reagent. The injected materials in the blank group were replaced with an equal volume of sterile water instead of plasmids. As shown in Fig. S3, the in vivo-jetPEI reagent did not affect the auditory function and morphology of cochlear cells in mice.

Two days after the injection, we detected the expression of the transfected gasdermin protein in the cochlea, mainly in the hair cells and supporting cells, but not in spiral ganglion neurons. This lack of expression may be related to the transfection time or specificity of transfection reagents (Fig. S4a). GSDME-WT and GSDME-MT exhibited similar expression patterns and levels (Fig. S4b). We also observed that some of the transfected gasdermin proteins co-localized with native DFNA5 (Fig. S5a-c) or mitochondria (Fig. S5d).

Four weeks after the injection, the ABR and DPOAE were assessed. We found that mice injected with *GSDME-MT* exhibited severe hearing impairment with significantly elevated ABR thresholds for click and tone-burst stimuli at all frequencies compared to those of the control group (Fig. 2b, c). Meanwhile, the mice injected with *GSDME-WT* maintained normal hearing at thresholds close to those of the control group. In addition, the DPOAE thresholds were significantly higher in the *GSDME-MT* group, but there were no differences between the *GSDME-WT* and control groups (Fig. 2d). The results of ABRs reflected the abnormal activity of the auditory afferent pathway, and DPOAE responses reflected the destruction of the outer hair cells (OHCs) due to the injection of *GSDME-MT* plasmids.

### *GSDME* mutations result in cell loss in the organ of Corti and spiral ganglion neurons

**Fig. 3:**
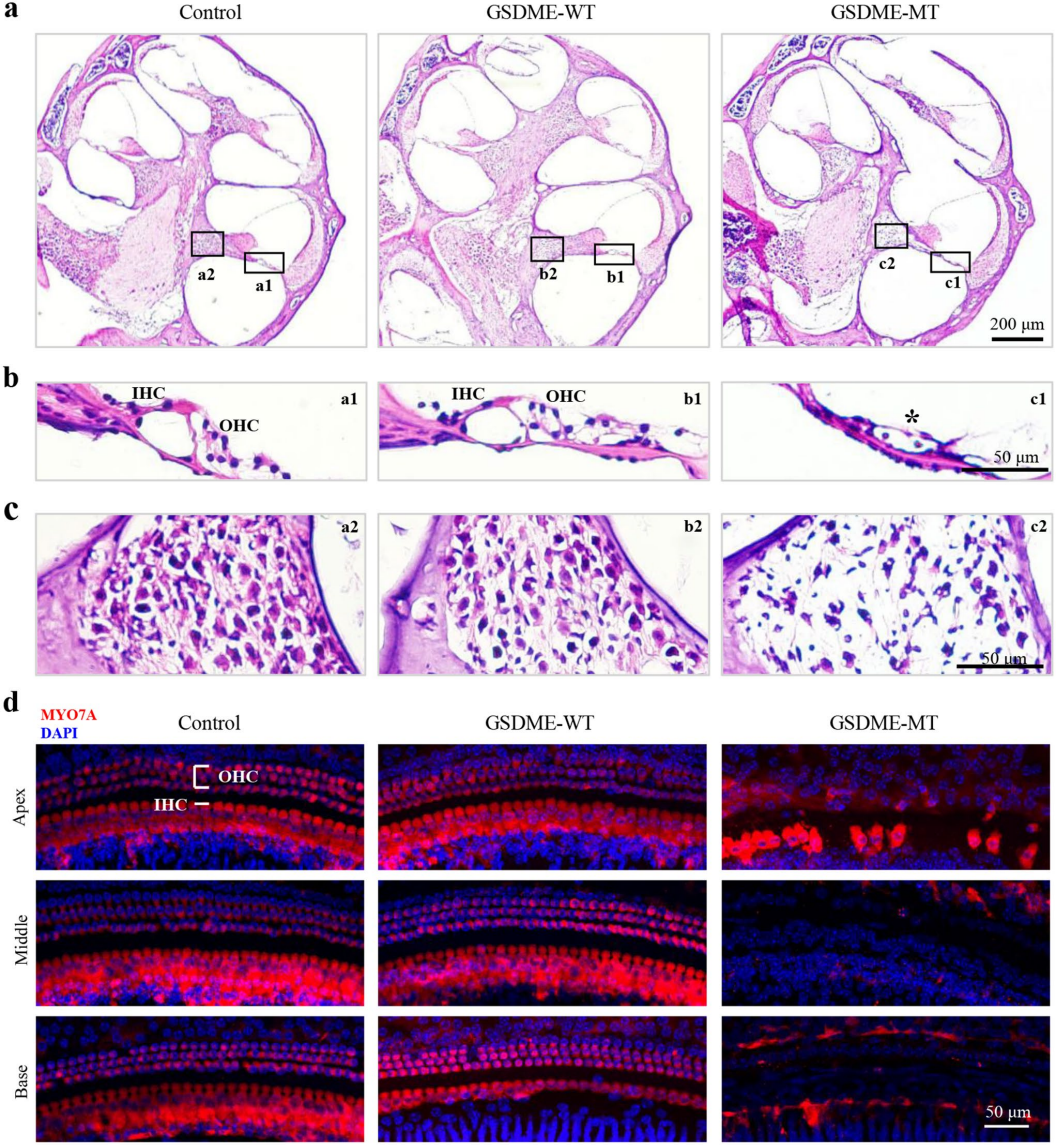
Cochlear morphology. (**a-c**) Representative images of the hematoxylin and eosin staining of the frozen cochlear sections showing the structure of the organ of Corti (**b**) and spiral ganglion neurons (**c**) in the basal turn. The asterisks indicate abnormalities of the organ of Corti in the basal turn of mice injected with *GSDME-MT* plasmids. (**d**) The hair cells in the apical, middle, and basal turns of the cochlea were immunolabeled for MYO7A (red). Mice injected with *GSDME-MT* plasmids lacked IHCs and OHCs in the basal and middle turns of the cochlea, and only a few IHCs survived in the apex turn. The scale bars represent 200 μm (**a**) and 50 μm (**b-d**). IHCs, inner hair cells; OHC, outer hair cells; MT, mutant

To assess the integrity of the principal structures of the cochlea, we evaluated the general cochlear cytoarchitecture of the mice after the assessments of the ABR and DPOAE. As shown in Fig. 3a, the organ of Corti, responsible for the mechano-transduction, amplification, and tuning of sounds, showed clear differences between the *GSDME-MT* and control groups. A closer evaluation of the inner hair cells (IHCs) and OHCs of the organ of Corti at the basal turn of the cochlea showed that the mice injected with *GSDME-MT* plasmids presented with a flat epithelium at the basilar membrane and complete loss of IHCs and OHCs (Fig. 3b).

Furthermore, we examined the spiral ganglion neurons that connect hair cells to the central auditory pathway. Loss of spiral ganglion neurons (Fig. 3c) was observed in the basal cochlear turn of the mice injected with *GSDME-MT* plasmids, but not in the control group. No evident structural abnormalities of the organ of Corti and spiral ganglion neurons were observed in the control mice. The structure of the stria vascularis and spiral ligament were normal in all three groups.

To further investigate the arrangement and number of hair cells, the cochlear basilar membranes of mice in all groups were dissected, and cochlear hair cells were visualized using immunofluorescence experiments. This analysis confirmed that mice injected with *GSDME-MT* plasmids lacked IHCs and OHCs in the basal and middle turns of the cochlea, and only a few IHCs survived in the apex turn. However, no abnormal arrangement, nor absence, of hair cells was observed in the control and *GSDME-WT* groups, except for a few OHCs that were lost in the basal turn in the *GSDME-WT* group (Fig. 3d).

### *GSDME* mutations induce pyroptosis and apoptosis

**Fig. 4:**
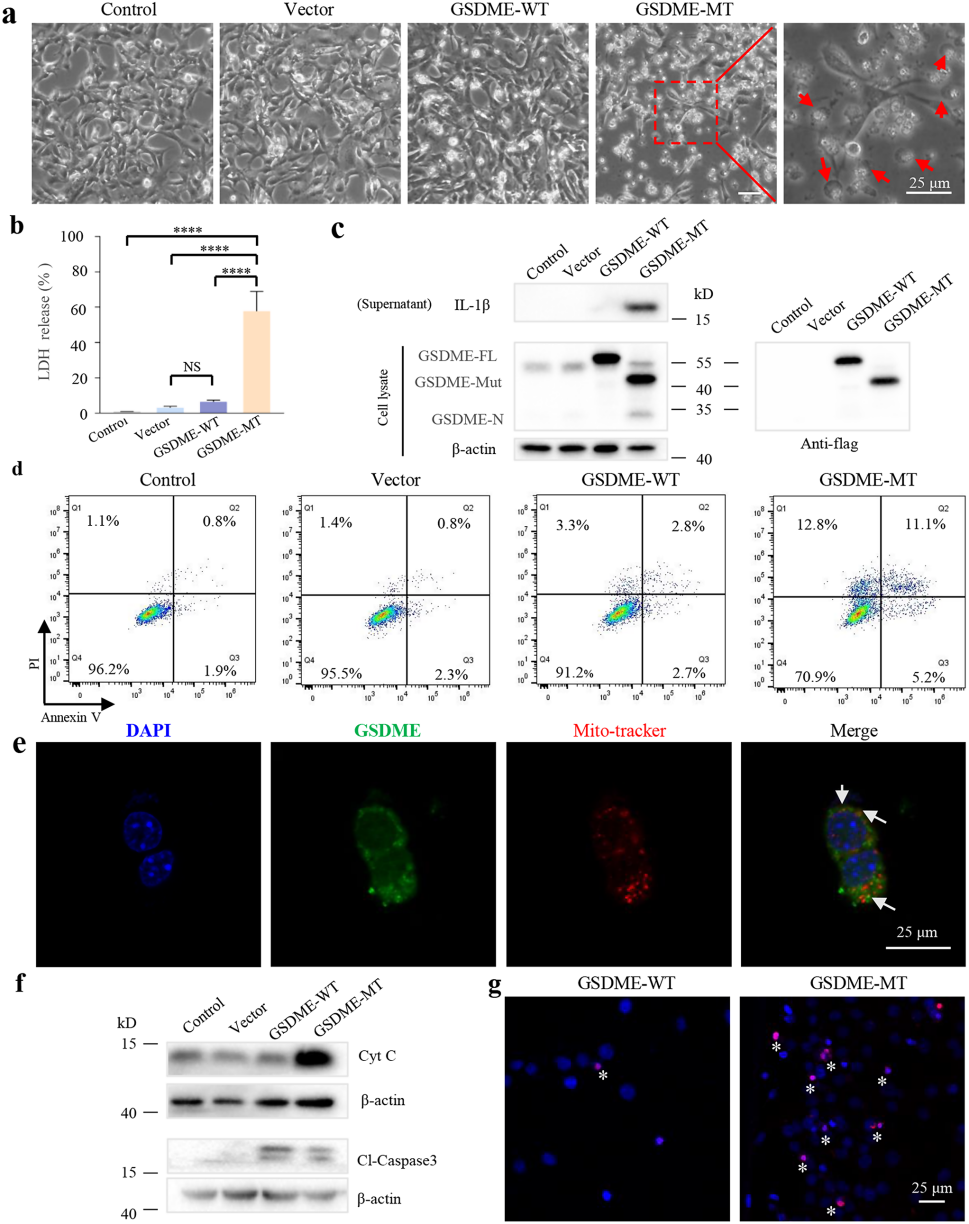
*GSDME* mutants induce pyroptosis and apoptosis. (**a**) Representative microscopic images of the HEI-OC1 cells 48 h post-transfection. Red arrowheads mark swollen cells. (**b**) Cytotoxicity measured by lactate dehydrogenase (LDH) release in the culture supernatants of HEI-OC1 cells 48 h post-transfection. (**c**) Immunoblots of *GSDME* in cell lysates and IL-1β released in culture media (supernatants). (**d**) Flow cytometry analysis of propidium iodide (PI) and Annexin V-FITC stained HEI-OC1 cells after transfection. (**e**) Confocal imaging of MitoTracker Red-stained HEI-OC1 cells after transfection of the *GSDME-MT* plasmids. The green channels show the expression of *GSDME*, whereas the red channels show mitochondrial staining with MitoTracker Red. The arrows indicate the co-localization of *GSDME* with MitoTracker red. **(f)** Immunoblots of Cyt C and cleaved caspase-3 (cl-caspase3) in cell lysates. **(g)** TUNEL stained assay. Asterisks indicate TUNEL-positive cells. Results are representative of at least three independent experiments. Control: no transfection; Vector: transfection with pcDNA3.1 plasmids; GSDME-WT: transfection with GSDME wild-type plasmids; GSDME-MT: transfection with GSDME mutant plasmids; GSDME-FL: full-length GSDME; GSDME-Mut: GSDME mutant; GSDME-N: N-terminal of GSDME; NS: no significant difference, ****: *p* < 0.0001. Scale bar, 25 μm

To further elucidate the molecular mechanism of deafness caused by *GSDME* gene mutation, *GSDME-MT* or *GSDME-WT* plasmids were transfected into HEI-OC1 cells. First, the changes in cell morphology were observed. As shown in Fig. 4a, cells transfected with GSDME-MT plasmids exhibited the classic characteristics of pyroptosis, such as large bubbles emerging from the plasma membrane and cell swelling. Concurrently, HEI-OC1 cells transfected with *GSDME-MT* plasmids displayed a significant increase in LDH release (Fig. 4b), further indicating plasma membrane rupture and leakage. In pyroptotic cells, pores can be formed in the cell membrane, allowing small-molecular-weight molecules, such as propidium iodide (PI), to enter and stain cells(Vande Walle andLamkanfi. 2016). *GSDME-MT* cells had a significantly higher proportion of PI-positive cells than those in the other groups, according to the flow cytometry analysis shown in Fig. 4d. Moreover, we examined the level of cytokines, such as IL-1β, and found an extensive release of IL-1β into the culture media (Fig. 4c), suggesting that *GSDME* mutations can trigger pyroptosis.

To elucidate how the *GSDME* mutation triggers pyroptosis, the level of N-terminal G*SDME* (GSDME-N), which is the key domain that triggers pyroptosis, was examined through western blotting. As shown in Fig. 4c, GSDME-N release was observed in cells expressing the *GSDME* mutation (GSDME-Mut), whereas the cleavage of GSDME into GSDME-N was completely absent in the cells expressing wild-type *GSDME* and those of the other two control groups. Altogether, our results suggest that the transient expression of *GSDME* mutants in HEI-OC1 cells induced pyroptosis.

In a previous study, GSDME-N was found to permeabilize the mitochondrial membrane, releasing Cyt C to augment mitochondrial apoptosis (Rogers et al. 2019). In HEI-OC1 cells transfected with *GSDME-MT* plasmids, we investigated whether GSDME-N targeted and permeabilized mitochondria to release apoptotic factors such as Cyt C. As shown in Fig. 4e, there were overlaps between the GSDME-N puncta and MitoTracker Red-stained mitochondria in *GSDME* mutation-expressing HEI-OC1 cells, indicating that GSDME-N targeted the mitochondria. Moreover, the protein levels of Cyt C and cleaved caspase3 (Cl-Caspase3) in the cell lysate were increased (Fig. 4f), confirming the rupture of the mitochondrial and plasma membranes. Flow cytometry analysis also demonstrated that transfection with *GSDME-MT* plasmids increased the percentage of annexin V-positive cells. Furthermore, positive TUNEL signals were detected in the HEI-OC1 cells transfected with GSDME-MT plasmids (Fig. 4g). We also detected TUNEL-positive cells in mice two days after injection of the *GSDME-MT* plasmids (Fig. S6). These results indicate that in addition to its function of triggering pyroptosis, GSDME-N released from the *GSDME* mutants can also contribute to mitochondrial permeabilization and augment the mitochondrial apoptotic pathway.

### Disulfiram and DMF mitigate the cytotoxicity of *GSDME* mutants

Our previous results indicated that GSDME mutants can trigger pyroptosis and apoptosis in HEI-OC1 cells; therefore, we further aimed to discover an approach to mitigate their cytotoxicity. GSDME-driven pyroptosis is regulated by post-translational modification (Hu et al. 2020b; Rogers et al. 2019), suggesting that the modification of the N-terminal of gasdermin proteins might be a drug target for mitigating pyroptotic cell death. We used DMF (20 and 50 µM) and disulfiram (1 and 5 µM), which are inhibitors of gasdermin pore formation, to investigate strategies for mitigating the cytotoxicity of GSDME mutants.

**Fig. 5:**
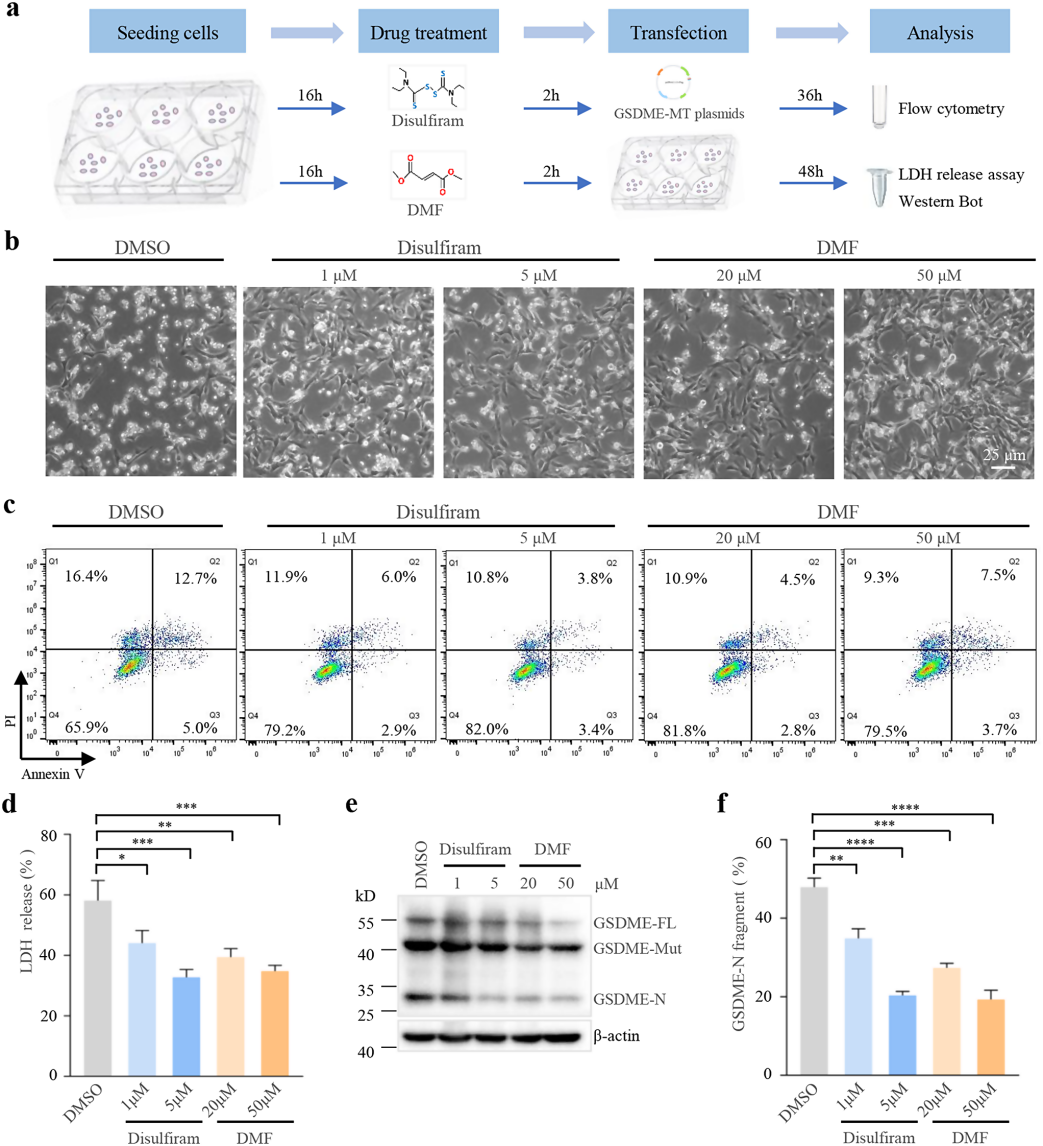
Disulfiram and DMF mitigate the cytotoxicity of GSDME mutants. (**a**) Timeline of drugs treatments and assays. (**b**) Microscopic images of HEI-OC1 cells expressing *GSDME* mutant untreated (DMSO) or treated with disulfiram or DMF as indicated. (**c**) Flow cytometry of propidium iodide and Annexin V-stained cells. (**d**) Assay of lactate dehydrogenase (LDH) release in the culture supernatants of HEI-OC1 cells expressing *GSDME* mutant untreated (DMSO) or treated with disulfiram or DMF. (**e**) Immunoblots of *GSDME* and β-actin in cell lysates of HEI-OC1 expressing *GSDME* mutant untreated (DMSO) or treated with disulfiram or DMF as indicated. (**f**) Quantitative analyses of GSDME-N release. Results are representative of at least three independent experiments performed in duplicate or triplicate. Mut, mutant; FL, full length. Error bars represent S.D., **p < 0.005, ***p < 0.0001, ****p < 0.00001

Drug treatments and assays for pyroptosis were performed according to the timeline presented in Fig. 5a. Morphological changes were observed in the HEI-OC1 cells transfected with *GSDME-MT* plasmids upon DMF or disulfiram exposure. As shown in Fig. 5b, the proportion of cells exhibiting pyroptotic morphological characteristics decreased in the drug-treated group compared to that in the control group treated with 0.1% dimethyl sulfoxide. Furthermore, flow cytometry analysis demonstrated that the proportion of Annexin V-positive and/or PI-positive cells decreased in the cells treated with disulfiram or DMF (Fig. 5c). We also found that the release of LDH was significantly decreased in the groups treated with disulfiram or DMF compared to that in the control group (Fig. 5d). Thus, treatment with disulfiram or DMF significantly inhibited pyroptosis induced by the GSDME mutants. Moreover, western blotting analysis showed that treatments with disulfiram or DMF resulted in a visible decrease in N-terminals cleaved from *GSDME* mutants (Fig. 5e, f), suggesting that disulfiram or DMF can inhibit pyroptosis by inhibiting the release of GSDME-N from the cleavage of *GSDME* mutants.

## Discussion

*GSDME* is a gene associated with autosomal dominant nonsyndromic sensorineural HL (OMM#600,994), whose phenotypes include late-onset, bilateral, symmetrical, and high-frequency sensorineural loss with progression, encompassing all frequencies. Several variants of *GSDME* have been described in population databases, but only those causing the skipping of exon 8 have been associated with HL (Bischoff et al. 2004; Cheng et al. 2007; Li-Yang et al. 2015; Mansard et al. 2022; Nishio et al. 2014; Park et al. 2010; Van Laer et al. 1998; Yu et al. 2003). The known pathogenic mutations c.991 − 15_991-13delTTC and c.1183 + 4 A > G of GSDME were identified in this study. The deleterious impact of the c.1183 + 4 A > G variant was confirmed through the assessment of the proband’s transcripts for the first time, similar to the impact of c.991 − 15_991-13delTTC mutations demonstrated in a previous study (Wang et al. 2018). By skipping exon 8, a premature stop codon is introduced, resulting in a protein with a truncated C-terminus. A previous study showed that the expression of human mutant *GSDME* was linked to programmed cell death in human cell lines and yeast; however, the mechanism was unclear (Van Rossom et al. 2015). Furthermore, Van Laer et al. (2005) proved that *GSDME* knockout mice did not differ from wild-type mice in their hearing thresholds, yet their fourth-row OHC count differed (Van Laer et al. 2005). Taken together, these studies suggest that the gain-of-function of *GSDME* mutants, from skipping exon 8, may be associated with HL.

To prove this hypothesis, we delivered plasmids expressing the *GSDME* mutant into the cochleae through round window injection. We found that the G*SDME* mutants resulted in HL, loss of IHCs and OHCs in the organ of Corti, and the degeneration of spiral ganglion neurons in the injected mice. Our findings are similar to those of a previous study regarding histopathology in a patient with a *GSDME* pathogenic variation, which resulted in the loss of IHCs and OHCs and severe degeneration of the stria vascularis, spiral ligament, and spiral ganglion throughout the cochlea (Nadol et al. 2015). Inner ear lesions in mice tend to be more severe than those observed in humans, potentially owing to the following reasons. First, we assessed the hearing of mice one month after injection and could not observe the process ofchanges. Second, the expression of the transfected plasmids in the cochlea can be challenging to control. Finally, in human patients carrying mutations in this gene, hearing changes tend to be age-related. Notably, we found that hair cells in the basal turn of the mouse cochlea were more severely damaged than those in the apical turn, probably attributable to the higher concentration of plasmids accumulated in the basal turn resulting from the round window injection technique.

To further elucidate the molecular mechanism underlying the deafness caused by mutations in *GSDME*, we transfected *GSDME* mutants into HEI-OC1 cells. The cells transfected with *GSDME-MT* plasmids exhibited swelling features, PI-positive staining, and increased release of IL-1β and LDH, suggesting that GSDME mutants triggered pyroptosis. In recent years, pyroptosis has been redefined as a process of programmed cell death mediated by gasdermin proteins. The gasdermin family proteins are encoded by six paralogous genes: *GSDMA*, *GSDMB*, *GSDMC*, *GSDMD*, *GSDME*, and *PJVK* (DFNB59). The NT and C-terminal (CT) domains of gasdermins are highly conserved and separated by a variable linker (Lu et al. 2022). When the CT domain is folded back on the NT domain, it auto-inhibits pore formation, and when it is cleaved in the linker region, it reveals a perforating NT domain that induces pyroptosis (Ding et al. 2016). *PJVK* is a more distantly related GSDM family member with a truncated non-homologous CT domain, but it is unclear whether it possesses pore-forming activity. Similar to *GSDME*, *PJVK* is involved in hearing impairment (OMM#610,219); however, the clinical symptoms differ, and the inheritance pattern is recessive, rather than dominant (Delmaghani et al. 2006). Unlike *Gsdme*^–/–^ mice, *Pjvk*^–/–^ mice exhibit hearing impairment (Schwander et al. 2007), suggesting that these mutants cause deafness through different mechanisms.

**Fig. 6:**
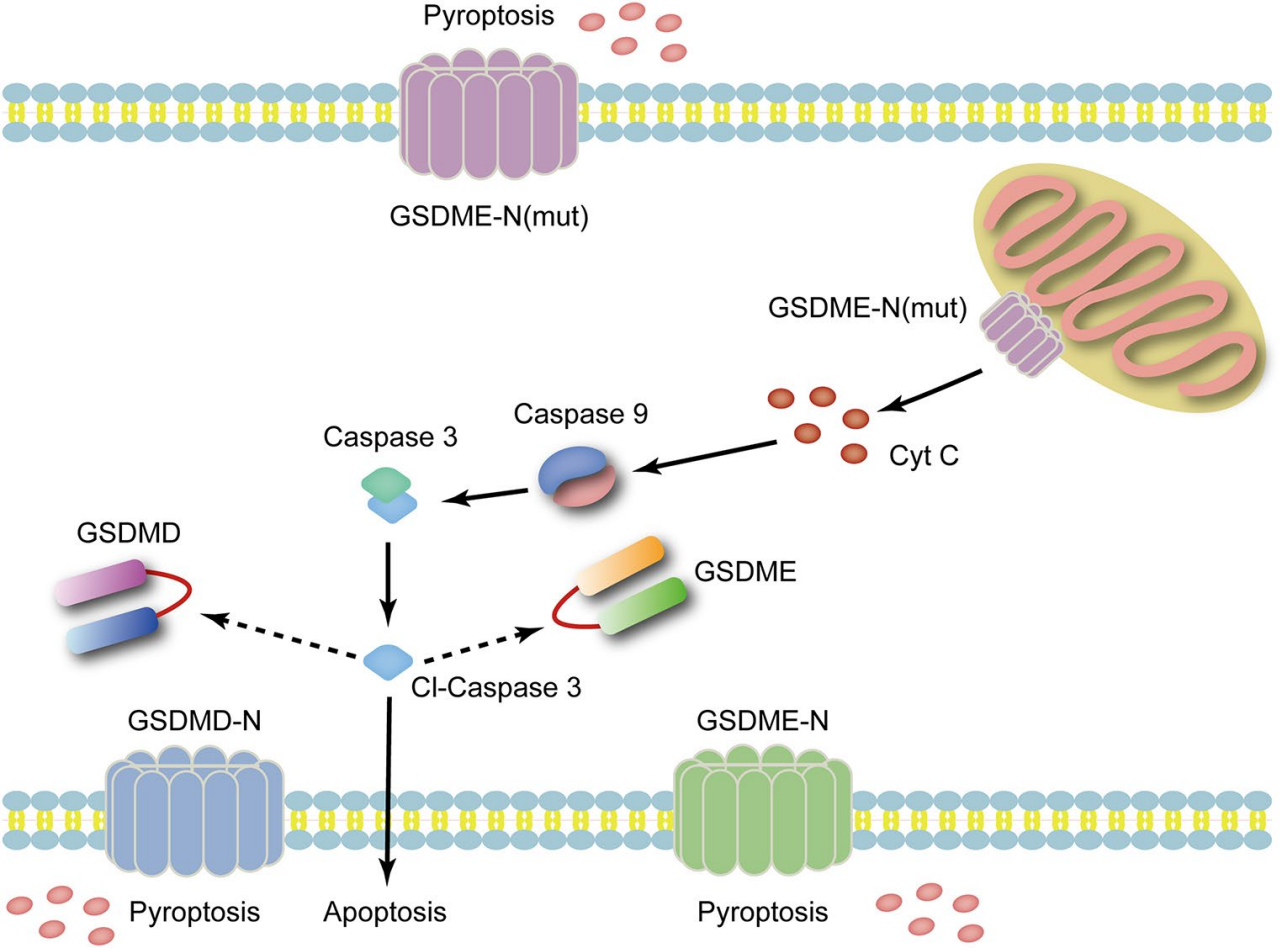
A model representing how a *GSDME* mutant triggers pyroptosis and apoptosis. The N-terminus liberated from the *GSDME* mutant permeabilizes the plasma and mitochondrial membranes. The N-terminus with pore-forming activity leads to pyroptosis and permeabilizes the mitochondria to release Cyt C and augments caspase-3 activation and apoptosis. The activation of caspase-3 may generate positive feedback on the cleavage of *GSDME*-*WT* and *GSDME-MT.* WT, wild-type; MT, mutant

The protein encoded by *GSDME* includes a pore-forming N-terminal domain (amino acids 1 to 270) and an autoinhibitory CT domain (amino acids 271 to 496). In the full-length GSDME protein, the CT domain folds back on the NT domain to auto-inhibit pore formation (Ding et al. 2016). Researchers believe that caspases are responsible for pyroptosis (Bergsbaken et al. 2009); however, recent studies have shown that cellular pyroptosis occurs in a caspase-independent manner (Shi et al. 2017). Some studies have shown that when the connection between the two domains is cleaved by the apoptotic protease caspase-3 or killer cell granzyme B (GzmB) specifically after Asp270, the self-inhibitory structure is destroyed, resulting in the release of the NT domain to activate the pore-forming properties and ultimately trigger pyroptosis (Wang et al. 2017; Zhang et al. 2020). Upon skipping exon 8, *GSDME* loses its self-inhibitory activity as its CT domain is destroyed. In our study, the release of the NT domain was observed after the expression of *GSDME* mutants in HEI-OC1 cells. However, whether this mutant is capable of pore-forming activity independent of cleavage by caspase-3 or GzmB remains unclear. GSDME-NT has also been shown to translocate to the mitochondrion, permeabilize the outer membrane, and disrupt mitochondrial function, leading to Cyt C release, which also activates caspase-3 and triggers overall cell death (Karmakar et al. 2020; Neel et al. 2023; Rogers et al. 2019). Similarly, our data indicated that *GSDME* mutants can form pores in the mitochondrion, resulting in the release of Cyt C and subsequently activating caspase-3 and, ultimately, apoptosis (Fig. 6). The activation of caspase-3 may generate a positive feedback loop that amplifies *GSDME* mutant cleavage, as recent findings have confirmed that the mitochondrial intrinsic apoptotic pathway can initiate *GSDME*-mediated pyroptosis (Lu et al. 2018; Rogers et al. 2017).

The above mechanism might explain how *GSDME* mutants induce HL, and the variable age of onset in humans suggests that other factors may induce the activation of pore-forming activity by *GSDME* mutants. The specific causative link between *GSDME* mutations and hearing impairment, while avoiding pathological damage to other organs where *GSDME* is highly expressed, remains unclear. *GSDME* is expressed in the developing and mature cochlea (Maeda et al. 2001), which have a limited ability to regenerate themselves; therefore, pyroptosis and/or apoptosis caused by *GSDME* mutants may result in progressive damage in the viability and function of cochlear hair cells, eventually leading to HL. Other tissues may be able to inhibit the expression of *GSDME* mutants or eliminate the unstable mutants in time. The deafness caused by *GSDME* mutations is post-lingual and progressive, suggesting that other factors may induce its pathogenicity, and the age of onset ranges from 10 to 50 years, which may differ from the time point of exposure to the incentives.

There are currently no efficient drugs or gene therapy methods to treat HL caused by *GSDME* mutations. Epigenetic mechanisms, such as DNA methylation (Kim et al. 2008) and post-translational modification (Hu et al. 2020a; Humphries et al. 2020; Rogers et al. 2019), are crucial targets for controlling GSDME-driven pyroptosis activity, suggesting that modification of the N-terminals of gasdermin proteins might be a drug target for resisting cell cytotoxicity. Rogers et al. (2019) further demonstrated that the phosphorylation of the residue Thr6 in *GSDME* blocks the activity of oligomerization and prevents membrane leakage, suggesting that it may play a crucial role in controlling pyroptotic activity (Rogers et al. 2019). An earlier investigation revealed that DMF could block pyroptosis by promoting succinate in *GSDME* at Cys45 and *GSDMD* at Cys191 to reduce inflammatory responses (Humphries et al. 2020). Moreover, disulfiram, an FDA-approved drug for treating alcoholism, has been shown to inhibit GSDMD pore formation by modifying the Cys191 residue of human *GSDMD* (Hu et al. 2020a). However, whether disulfiram also targets cysteine residues on GSDME-N remains unclear. Therefore, in this study, we selected disulfiram and DMF to investigate the cytotoxicity resistance of *GSDME* mutants and found that treatment with disulfiram or DMF significantly inhibited pyroptosis induced by *GSDME* mutants. The decrease in the generation of the N-terminals of *GSDME* mutants suggests that disulfiram and DMF can block pyroptotic activity by inhibiting the cleavage of *GSDME* mutants. However, the regulatory mechanism needs to be further elucidated in future research.

The present study elucidated the molecular mechanism associated with HL caused by GSDME gene mutation, offering novel insights for potential treatment strategies.

Our study had some limitations. We did not clarify the reason for the late onset of deafness and the mechanism by which disulfiram and DMF can alleviate the toxicity of mutant proteins, warranting future investigations. In future studies, we aim to establish a mouse model of *GSDME* gene point mutation to delve deeper into these aspects.

In conclusion, our findings provide comprehensive insights into the mechanism of HL caused by *GSDME* mutations. Mice injected with GSDME-MT expression plasmids showed both functional and morphological inner ear impairments, presenting significant evidence for the link between hearing impairment and *GSDME* mutation. Overall, we demonstrated that *GSDME* mutants induced pyroptosis and enhanced the mitochondrial apoptotic pathway in HEI-OC1 cells, triggered by the pore-forming GSDME-N released from GSDME mutants. We also found that treatment with disulfiram or DMF inhibited the cleavage of *GSDME* mutants, thereby blocking pyroptosis directly or indirectly. This study offers novel insights into the mechanisms and treatment of deafness caused by the cytotoxicity of *GSDME* mutants.

### Electronic supplementary material

Below is the link to the electronic supplementary material.

Supplementary Material 1

## Data Availability

The data that support the findings of this study are available from the corresponding authors upon reasonable request.
