# Supplementary material for: Gain-of-function variants in GSDME cause pyroptosis and apoptosis associated with post-lingual hearing loss
Source: Hum Genet. 2024 Jul 27;143(8):979–93. doi: 10.1007/s00439-024-02694-x (PMC11303571; doi:10.1007/s00439-024-02694-x)
Supplement: Supplementary file 1 — Supplementary Material 1 [file 439_2024_2694_MOESM1_ESM.pdf]

Supplementary Information

Gain-of-function variants in GSDME cause pyroptosis and apoptosis associated with post-lingual hearing loss

Yun Xiao, Lei Chen, Kaifan Xu, Meijuan Zhou, Yuechen Han, Jianfen Luo, Yu Ai, Mingming Wang, Yu Jin, Ruifeng Qiao, Shuhui Kong, Zhaomin Fan, Lei Xu*, Haibo Wang*

*Co-corresponding authors:

Lei Xu

Department of Otorhinolaryngology Head and Neck Surgery, Shandong Provincial ENT Hospital, Jinan, Shandong 250022, China

E-mail: sdphxl@126.com

Haibo Wang

Department of Otorhinolaryngology Head and Neck Surgery, Shandong Provincial ENT Hospital, Jinan, Shandong 250022, China

Fax: +86 531 68777588

E-mail: whboto11@163.com

Supplementary figures

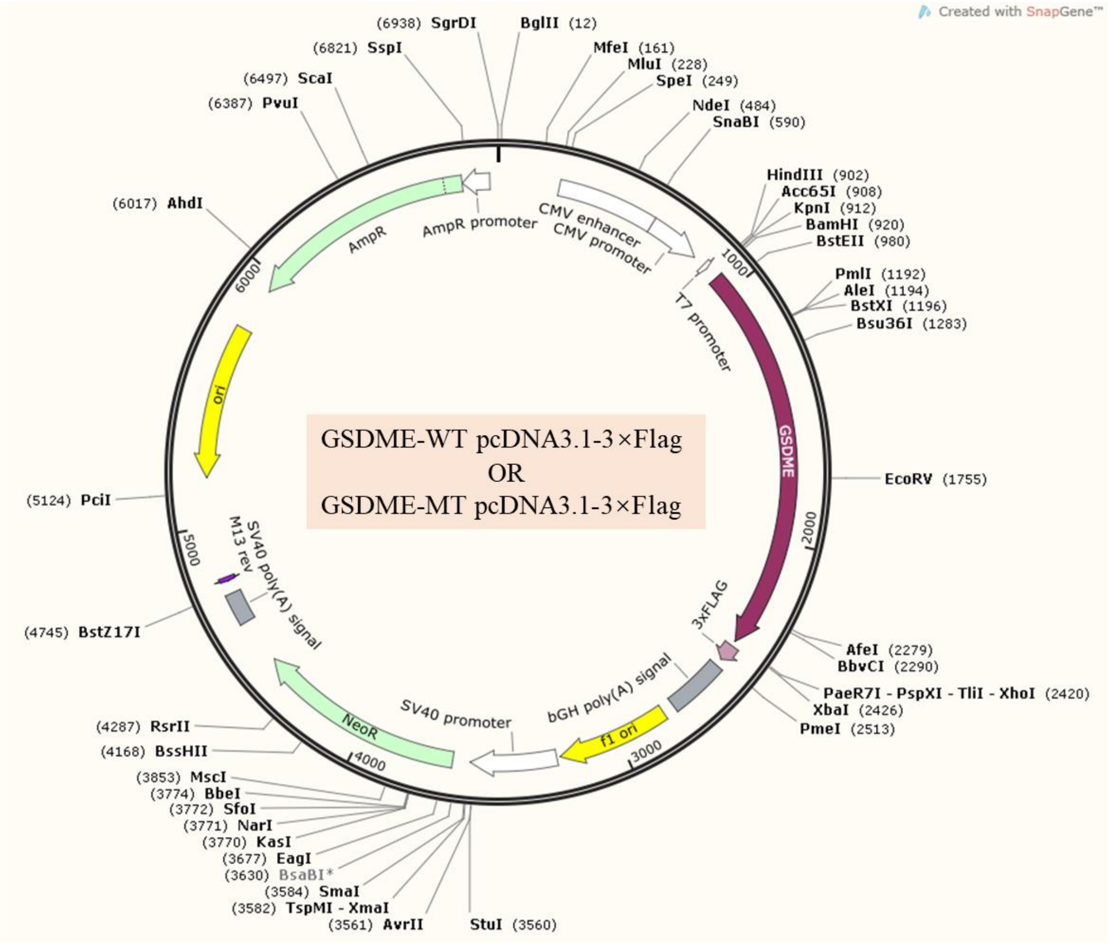

Fig. S1 Schematic diagram of the pcDNA3.1-3×Flag plasmids cloned with human *GSDME-WT* or *GSDME-MT* cDNAs

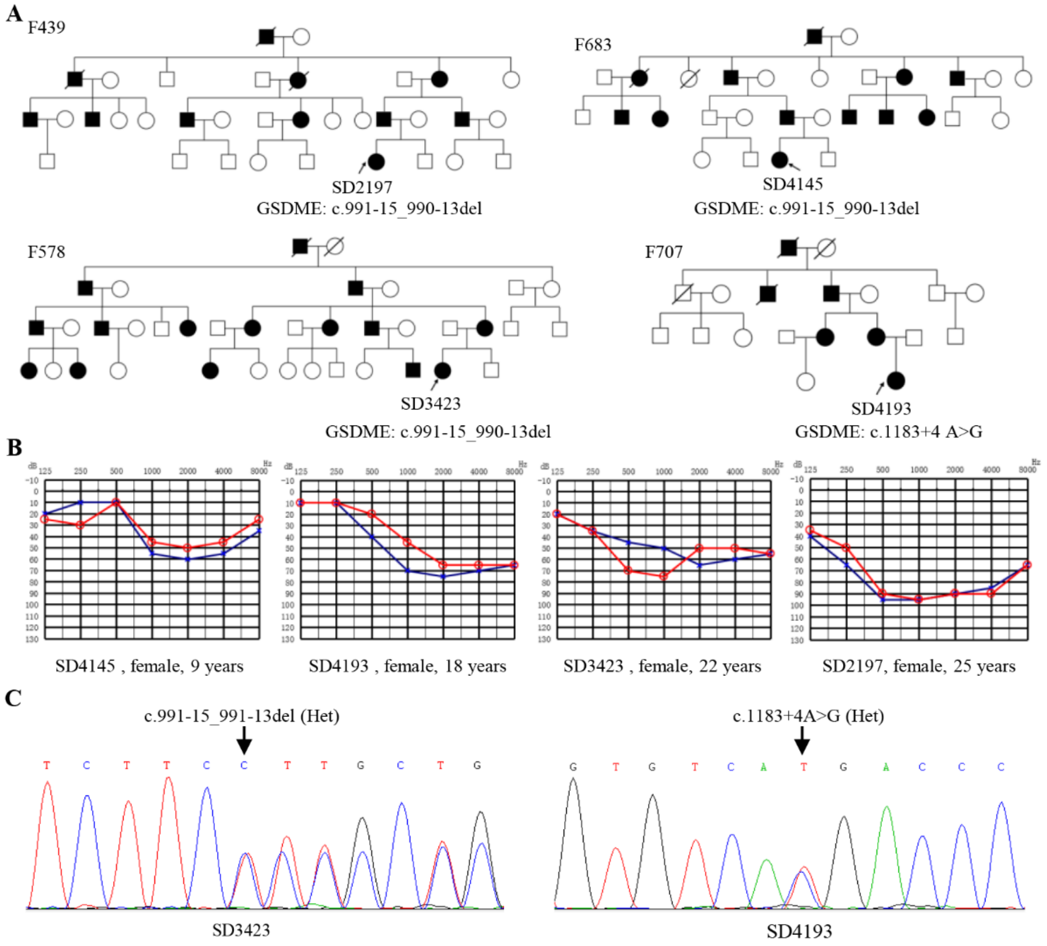

Fig. S2 Identification and validation of the *GSDME* mutations in the families with post-lingual hearing loss. **(A)** Pedigrees of families with post-lingual HL. Filled symbols for males (squares) and females (circles) represent affected individuals. The arrow denotes the proband. **(B)** Audiograms of the probands of the four families. Symbols “x” and “o” denote air conduction pure-tone thresholds at different frequencies in the left and right ears, respectively. dB, decibels; Hz, Hertz. **(C)** Representative DNA sequence chromatograms from probands SD3423 and SD4193, showing the GSDME mutations c.991-15_991-13del and c.1183+4A>G

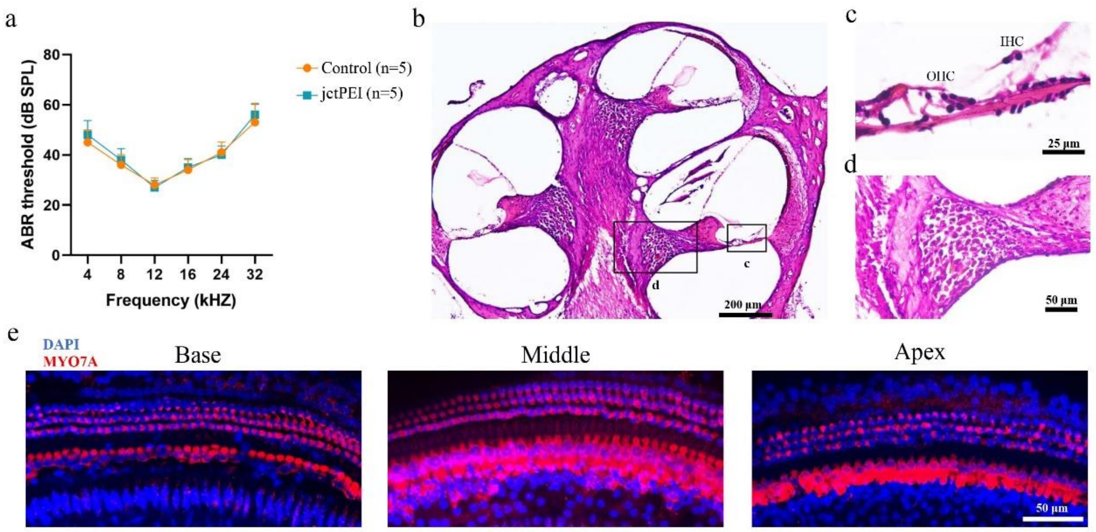

Fig. S3 Auditory functional test and cochlear morphology of the blank group. The injected materials in the blank group were replaced with an equal volume of sterile water instead of plasmids. The *in vivo*-jetPEI reagent had no effect on the auditory function and morphology of cochlear cells in mice. (**a**) Auditory functional tests were performed four weeks after the injection. (**b-d**) Representative images of the hematoxylin and eosin staining of the frozen cochlear sections showing the structure of the organ of Corti (**c**) and spiral ganglion neurons (**d**) in the basal turn. (**e**) The hair cells in the apical, middle, and basal turns of the cochlea were immunolabeled for MYO7A (red)

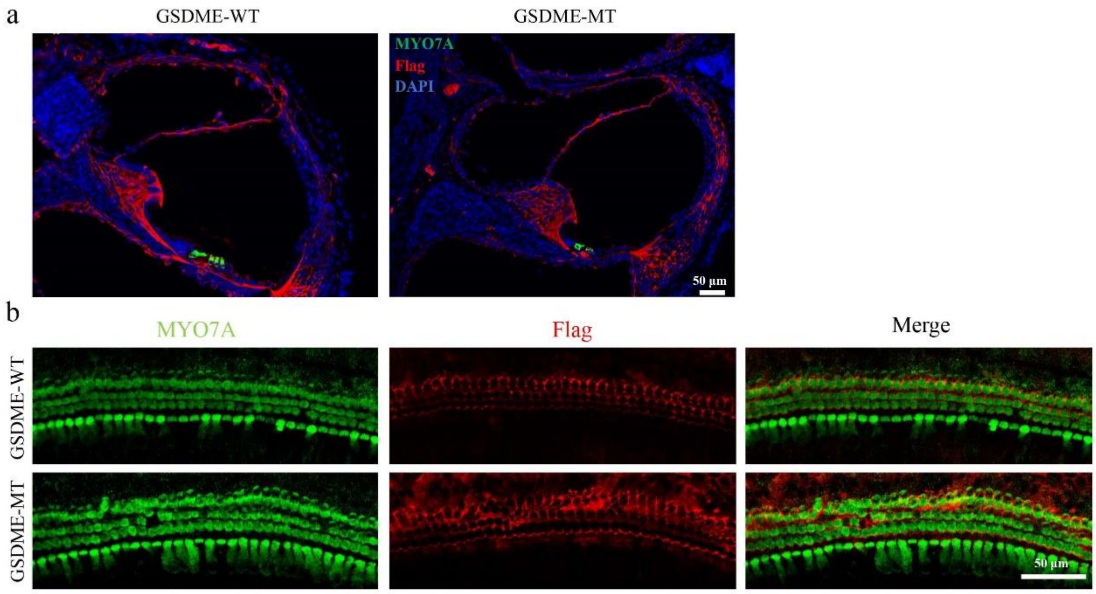

Fig. S4 Expression of transfected gasdermin protein. (**a**) Two days after the injection, the expression of transfected gasdermin protein could be detected in the cochlea. (**b**) GSDME-WT and GSDME-MT show similar expression patterns and levels. The green channels show hair cells stained with MYO7A, and the red channels show transfected gasdermin proteins stained with anti-flag

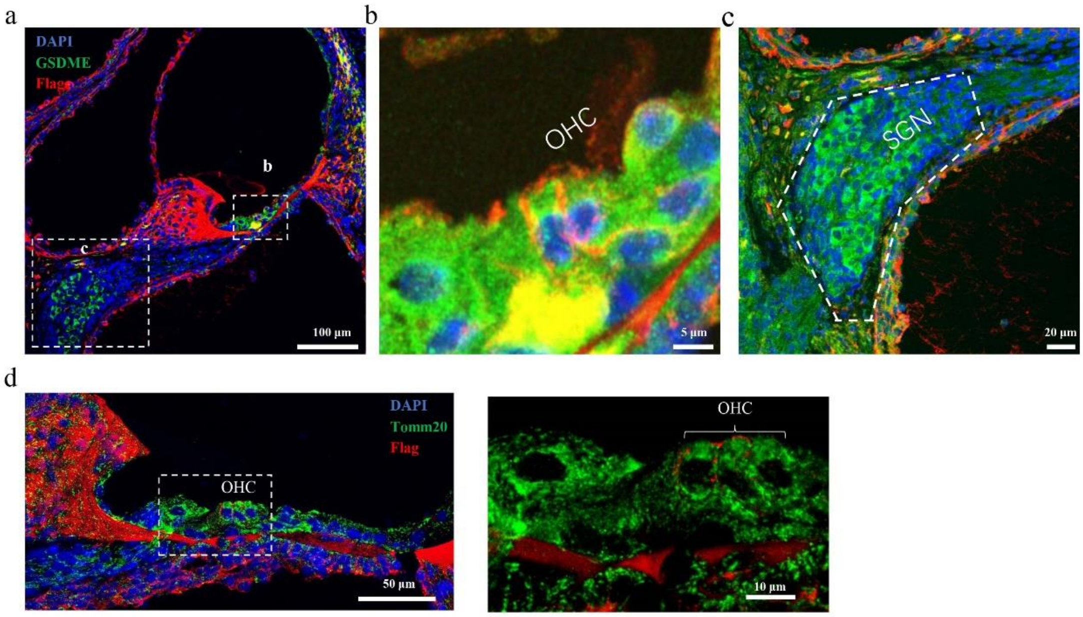

Fig. S5 Some of the transfected gasdermin proteins were co-localized with native DFNA5 or mitochondria. (**a-c**) The green channels show the expression of native DFNA5, whereas the red channels show transfected gasdermin proteins staining with anti-flag. (**d**) The green channels show mitochondrial staining with TOMM20, and the red channels show transfected gasdermin proteins stained with anti-flag. OHC, outer hair cell. SGN, spiral ganglion cell

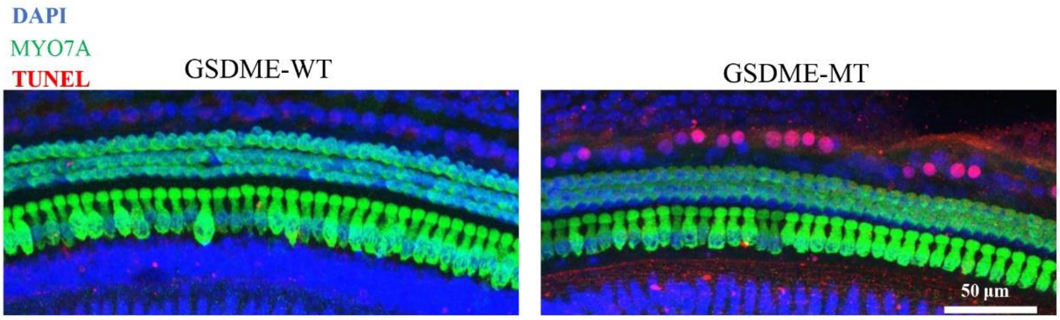

Fig. S6. TUNEL stained assay of the mice after 2 days injection. The red color indicates TUNEL-positive cells. GSDME-WT: injection with GSDME wild-type plasmids; GSDME-MT: injection with GSDME mutant plasmids
